# Colchicine in atrial fibrillation: are old trees in bloom?

**DOI:** 10.3389/fphys.2023.1260774

**Published:** 2023-10-17

**Authors:** Yujia Zhan, Honghua Yue, Xueshan Zhao, Juan Tang, Zhong Wu

**Affiliations:** ^1^ Department of Cardiovascular Surgery, West China Hospital, Sichuan University, Chengdu, China; ^2^ Acupuncture and Moxibustion School of Teaching, Hospital of Chengdu, University of Traditional Chinese Medicine, Tianjin, China; ^3^ Key Laboratory of Emergency and Trauma, Ministry of Education, Hainan Medical University, Haikou, China

**Keywords:** colchicine, atrial fibrillation, postoperative atrial fibrillation, inflammation, NLRP3 inflammasome

## Abstract

Colchicine is a widely used drug that was originally used to treat gout and rheumatic diseases. In recent years, colchicine has shown high potential in the cardiovascular field. Atrial fibrillation (AF) is a cardiovascular disease with a high incidence. One of the most frequent complications following cardiovascular surgery is postoperative atrial fibrillation (POAF), which affects patient health and disease burden. This article reviews the research status of colchicine in AF and summarizes the relevant progress.

## 1 Introduction

Colchicine is an ancient drug. It has been extensively used to treat gout and rheumatic diseases for centuries (Roubille et al., 2013). In recent years, colchicine has become a good candidate drug for cardiac protection and plays a variety of roles, including inhibiting arrhythmia (Imazio and Nidorf, 2021). Approximately one-quarter of adults may have Atrial fibrillation (AF) during their lifetime (Baman and Passman, 2021). One of the most frequent complications of cardiovascular surgery is POAF, which can result in heart failure and stroke (Echahidi et al., 2008). This article reviews the literature on colchicine and AF and summarizes the potential mechanism of its application in AF.

## 2 Background and pharmacology of colchicine

Colchicine is a microtubule disassembling agent that is extracted from cum autumnale and has anti-inflammatory properties (Deftereos et al., 2013a; Roubille et al., 2013). It is a widely accessible, reasonably priced medicine with good safety and few adverse drug reactions (Aimo et al., 2021a). Colchicine can inhibit microtubule polymerization and mitosis, stop neutrophils from becoming activated, degranulating, and migrating, and have anti-inflammatory effects (Finkelstein et al., 2010). Colchicine is frequently used to treat and prevent gout and rheumatic diseases, Behcet’s disease, pericarditis and familial Mediterranean fever (FMF) (Terkeltaub, 2009).

The bioavailability of colchicine is approximately 44%, and it is absorbed through the digestive system and reaches its highest plasma concentration in less than 1 h (Deftereos et al., 2013b). Colchicine binds to albumin and undergoes glucuronidation and cytochrome CYP3A4 metabolism in the liver (Hemkens et al., 2016). Approximately 10%–20% of the drug is cleared by P-glycoprotein (P-gp) transporters in the kidney (Hemkens et al., 2016). Therefore, P-glycoprotein inhibitors or potent CYP3A4 inhibitors cannot be used in conjunction with colchicine. The half-life of colchicine in healthy volunteers is 27–31 h (Pascart and Richette, 2018). After the termination of treatment, the biological effect lasts 24–48 h, and the final elimination half-life is 16 h. The half-life might be two or three times longer in people with renal or hepatic dysfunction (Phelps, 1970).

Symptoms of the digestive system, including nausea, vomiting, and diarrhoea, are the most frequent adverse effects of colchicine (Abrantes et al., 2021). Excessive serum concentrations may lead to serious adverse reactions such as muscle toxicity or cytopenia (Pascart and Richette, 2018). Currently, numerous studies have demonstrated the safety of administering low-dose colchicine over an extended period of time in patients with healthy liver and kidney function or long-term use of 0.5–1.0 mg colchicine in patients with FMF, gout, pericarditis and coronary heart disease (Ozen et al., 2016; Imazio et al., 2020; Nidorf et al., 2020; Sammaritano et al., 2020; Andreis et al., 2021a). However, a few small case reports have reported the toxicity of colchicine, such as neuromyopathy (Altiparmak et al., 2002). Therefore, colchicine toxicity should be determined even in patients receiving long-term standard doses of colchicine. At present, a specific antidote of colchicine has been developed for use in preclinical trials (Eddleston et al., 2018). In addition, colchicine is lipophilic, can cross the placenta and is eliminated in breast milk; however, research has shown that taking colchicine while pregnant has no impact on the risk of miscarriage or foetal deformity (Brucato et al., 2019).

## 3 Use of colchicine in cardiovascular disease

Colchicine was first used 40 years ago to demonstrate the role of antimicrotubule proteins as anti-atherosclerotic agents, and since then, LoDoCo has been proposed for the treatment and prevention of recurrent pericarditis initially (Chaldakov, 1982; Chaldakov, 2018; Andreis et al., 2021b). The unique properties of colchicine give it remarkable potential in cardiovascular diseases such as pericardial syndrome recurrence, atherosclerotic plaque inflammation, atrial fibrillation recurrence and ventricular remodelling (Andreis et al., 2021a). With continued research progress, colchicine has been used to prevent coronary atherosclerosis, POAF, and heart failure (Imazio and Nidorf, 2021). A meta-analysis of 22 studies determined the effectiveness and safety of a low dose (0.5 mg daily) of colchicine given for a long period of time (greater than 6 months) to treat cardiac disease (Schattner, 2022). This study further confirmed the role of colchicine in reducing the risk of arrhythmia recurrence, recurrent pericarditis, cardiogenic death, stroke, coronary revascularization or hospitalization, and myocardial infarction (Schattner, 2022). Schattner et al. systematically reviewed and summarized 126 RCTs published in the past 20 years. These studies supported the anti-inflammatory effects of colchicine in the treatment and prevention of acute pericarditis, postcardiac injury syndrome after cardiac surgery, POAF, acute coronary syndrome and stable coronary artery disease (Guindo et al., 1990). Furthermore, most studies have shown that in addition to diarrhoea and myalgia, which may lead to drug withdrawal, there are almost no serious adverse events related to colchicine, which confirms the safety of LoDoCo (Guindo et al., 1990).

Colchicine is a second-line therapeutic option for pericarditis (Dainese et al., 2011; Deftereos et al., 2013b). Adding colchicine to standard treatments can reduce the recurrence rate of patients with pericarditis. Additionally, colchicine can be used to treat postoperative recurrent pericardial effusion and reduce the incidence of postoperative pericardial incision syndrome (Imazio et al., 2013a; Verma et al., 2015; Imazio and Nidorf, 2021). Colchicine decreased the incidence of recurrent pericarditis, pericardial incision syndrome following surgery and perioperative AF in an RCT by Verma et al. (Imazio et al., 2014). The COPPS-2 study by Imazio et al. obtained the same conclusion in terms of postoperative pericardial incision syndrome, but the incidence of POAF or postoperative pericardial/pleural effusion was not significantly decreased by colchicine (Robinson et al., 2022).

Long-term LoDoCo can be used for secondary prevention of ischaemic stroke and myocardial infarction (Tardif et al., 2019). The COLchicine Cardiovascular Outcomes Trial (COLCOT) of 4,745 patients demonstrated that a daily dose of 0.5 mg of colchicine to treat patients with myocardial infarction within 30 days could effectively reduce ischaemic cardiovascular events (Bo et al., 2020). Using LoDoCo as soon as possible after myocardial infarction can significantly reduce the incidence of ischaemic cardiovascular events (Hennessy et al., 2019). Studies have also demonstrated that low-dose colchicine was safe and tolerable after acute myocardial infarction (Deftereos et al., 2015). Colchicine has been proven in systematic reviews to lower the risk of myocardial infarction, but it has no discernible impact on all-cause mortality (Hemkens et al., 2016). In patients with myocardial infarction, colchicine can decrease the infarct area and the concentration of the creatine kinase muscle-brain (CK-MB) fraction in cardiac MRI (Bresson et al., 2021; Li et al., 2022). Furthermore, colchicine can inhibit cardiac inflammation after acute myocardial infarction and reduce cardiac remodelling (Sgouropoulou et al., 2019). Low-dose colchicine treatment, however, did not appreciably lower C-reactive protein levels within 30 days of acute myocardial infarction (Deftereos et al., 2015). Studies have shown that the cardiovascular effect of colchicine may not be related to its anti-inflammatory effect (Kukuy et al., 2017; Grajek et al., 2021).

A number of meta-analyses have demonstrated the positive effects of LoDoCo on the prognosis of patients with coronary heart disease, such as a significant reduction in cardiovascular mortality, myocardial infarction, and stroke (Samuel et al., 2021a; Samuel et al., 2021b; Xia et al., 2021; Ma et al., 2022). The systematic review by Samuel et al. also supported this result (Fiolet et al., 2021). However, after administering colchicine, there was no discernible difference in all-cause mortality (Nidorf and Thompson, 2007). Colchicine has been associated with a reduction in inflammation in people with stable coronary disease and has been proven to have antiatherosclerotic properties (Wójcicki et al., 1986; Akrami et al., 2021).

Taken regularly, low-dose colchicine can greatly improve the survival rates of individuals with acute and chronic coronary syndrome and reduce the frequency of serious adverse cardiac events (Giannopoulos et al., 2015; Imazio et al., 2020). The overall safety is satisfactory, and its therapeutic and preventive value for chronic coronary artery disease has been recognized to some extent (Aimo et al., 2021b). A meta-analysis that included 12 studies confirmed its effects. Colchicine decreased the risk of cardiovascular events in CCS patients by approximately 50% and the risk of ACS by 23% compared to the placebo (Yasgur, 2023). Not long ago a new pilot study demonstrated that colchicine could be added the day after PCI to reduce a patient’s risk of ischemic events and to mitigate the increased risk of aspirin-related bleeding (Papageorgiou et al., 2017).

However, the frequent complications such as gastrointestinal reactions caused by colchicine treatment often leads to withdrawal from the study, especially in postoperative patients (Salih et al., 2017; Andreis et al., 2021c; Robinson et al., 2022). The efficacy of colchicine is significantly affected by the interruption rate of treatment. A study confirmed that using colchicine to treat cardiovascular disease increased the risk of adverse gastrointestinal reactions and myalgia but has no significant relationship with other adverse reactions (Frommeyer et al., 2017). In addition, acute infusion of colchicine can increase the incidence of ventricular tachyarrhythmias in cardiac models (Kirchhof et al., 2016).

In summary, colchicine has been shown to prevent cardiovascular disease, but before routine use of colchicine in practice, more research is required to further explore the effectiveness and safety of colchicine and to find appropriate concomitant therapies to improve and reduce gastrointestinal complications.

## 4 Application and research of colchicine in AF

AF is a common arrhythmia with a prevalence of approximately 3% (Brundel et al., 2022). AF causes rapid irregular abnormal contraction of atrial myocardial cells, which leads to palpitations, dizziness and other symptoms (Zimetbaum, 2017). The most serious consequences of AF are heart failure and stroke (Baman and Passman, 2021). A number of variables, including age, smoking, obesity, hypertension, and diabetes, are linked to the occurrence of AF (Fuster et al., 2011). The main pathogenesis of AF is affected by many factors, such as blood pressure, atrial remodelling, increased autonomic nervous tension, inflammation, oxidative stress, and atrial electrical abnormalities (Frendl et al., 2014).

The use of colchicine to treat AF has been focused on the prevention of POAF. With an incidence of 20%–50%, POAF is the most prevalent arrhythmia after cardiovascular surgery (Echahidi et al., 2008; Haghjoo et al., 2012; Yadava et al., 2014). Definition, monitoring duration and technical methods can significantly affect the detection rate. POAF is generally considered to be a self-limiting disease, but data show that the occurrence of POAF is related to the incidence of stroke, acute renal infarction, congestive heart failure, and death (Raiten et al., 2015). As the population has aged over the past few years, there has been an increase in the number of elderly patients undergoing cardiac surgery, and the hospitalization time and medical burden of patients can increase (Imazio et al., 2011; Rezaei et al., 2020).

Age, race, and severe cardiovascular risk factors may contribute to POAF (Ishii et al., 2005). In addition, perioperative factors can also lead to POAF, such as pericarditis, autonomic nerve imbalance, catecholamine overdose or fluid transfer (Abdelhadi et al., 2004; Davis et al., 2010), which can change the atrial refractory period and create an environment that triggers the occurrence of AF (Echahidi et al., 2008; January et al., 2014; Rezaei et al., 2020).

At present, the main drugs used to prevent AF include β-blockers, antiarrhythmic drugs, cardiac glycosides and a variety of anti-inflammatory drugs. Generally, β-blockers are the first choice, and preoperative amiodarone can effectively lower the occurrence of AF. In the ACC/AHA guidelines, colchicine is recommended in Class IIb (Lu et al., 2016; Janu et al., 2019).

The recurrence of AF may be caused by atrial inflammation or atrial remodelling (Koyama et al., 2009; L et al., 2016). The role of colchicine in avoiding AF after cardiac surgery and reducing hospitalization time has been confirmed to some extent (Deftereos et al., 2014; Trivedi and Sadadia, 2014; Rezaei et al., 2020). The data showed that the use of colchicine for 3 months after ablation could reduce the possibility of AF recurrence by 37% after 15 months (Deftereos et al., 2012; Imazio et al., 2013b). Colchicine could considerably lower the incidence of postoperative pericardial effusion and POAF in the COPPS test and COPPS-POAF substudy (Rezaei et al., 2020; Shvartz et al., 2022a; Robinson et al., 2022). A double-blind RCT involving 240 subjects showed that short-term administration of colchicine after cardiac surgery significantly prevented POAF and systemic inflammation, but the incidence of diarrhoea and abdominal pain increased significantly (Zhao et al., 2022). A meta-analysis showed that colchicine could prevent the occurrence of POAF, its efficacy had no significant relationship with the prolongation of treatment time, and there were fewer adverse reactions (Lennerz et al., 2017). Another meta-analysis obtained similar results, but the use of colchicine significantly increased adverse gastrointestinal reactions (Ge et al., 2022). A number of meta-analyses have noted that colchicine could effectively prevent the occurrence of AF. Although it does not increase the incidence of major adverse events, it increases the likelihood of gastrointestinal side effects such as diarrhoea and abdominal pain. These side effects may lead to the interruption of treatment so that a complete therapeutic effect cannot be achieved. Therefore, further research on the dose and duration of colchicine is needed (Andreis et al., 2021c; Shvartz et al., 2022b; Kommu and Arepally, 2023). Some studies have shown that the prevalence of POAF may not be significantly impacted by colchicine because of the limited sample size (Zarpelon et al., 2016; Tabbalat et al., 2020). Clinical studies on the prevention of AF by colchicine in recent years are shown in Table 1. At present, there are 27 ongoing clinical trials for the use of colchicine in AF, of which 8 are under recruitment. The efficacy and safety of colchicine are expected to be further confirmed in the future.

**TABLE 1:** Clinical evidence on colchicine for the treatment of AF.

First author	Year	Study type	Study design	Colchicine dose and duration	Patients	Main conclusion
Tabbalat et al. (2016)	2020	Double-blind RCT	AF after cardiac surgery	1 mg 12–24 h before surgery and 0.5 mg qd until hospital discharge	172	No reduction in POAF
Gulick et al. (1989)	2016	Randomized trial (open label)	AF after cardiac surgery	0.5 mg bid, and 2 mg before surgery (half dose if < 70 kg) for 8 days (mean)	360	No reduction in POAF
Tabbalat et al. (2020)	2016	Randomized trial (open label)	AF after myocardial revascularization surgery	1 mg bid before surgery, and of 0.5 mg bid until hospital discharge	140	No reduction in POAF
Robinson et al. (2022)	2014	Double-blind RCT	AF after cardiac surgery	0.5 mg bid in patients ≥70 kg or 0.5 mg qd in patients <70 kg for 1 month	360	No reduction in POAF
Deftereos et al. (2012)	2014	Double-blind RCT	AF after pulmonary vein isolation in paroxysmal AF patients	0.5 mg bid for 3 months	223	Reduction in POAF
Imazio et al. (2013b)	2012	Double-blind RCT	AF after radiofrequency ablation treatment	0.5 mg bid for 3 months	161	Reduction in POAF
Rezaei et al. (2020)	2011	Double-blind RCT	AF after cardiac surgery	1.0 mg bid on postoperative Day 3 followed by 0.5 mg bid for 1 month in patients ≥70 kg, halved doses for patients <70 kg or intolerant to the highest dose	336	Reduction in POAF

## 5 Mechanism of colchicine in AF

### 5.1 Anti-inflammatory effects

Inflammation is one of the important pathogeneses of AF, and it is closely related to an increase in white blood cells (Davis et al., 2010). Inflammatory mediators play an important role in AF. Through different mechanisms, IL-6 can control cardiovascular function and alter the responsiveness of adrenergic receptors (Pagani et al., 1992), promote left ventricular remodelling (Yokoyama et al., 1993) or lead to myocardial systolic dysfunction (Wan and Yim, 1999). IL-8 can activate leukocytes and neutrophils (Chandrasekar et al., 1999), activate the proapoptotic pathway in endothelial cells, and continuously increase heart damage (Liu et al., 2007). Chronic inflammation, including elevated serum levels of C-reactive protein, IL-1β, IL-6, and tumour necrosis factor (TNF), can contribute to the development and maintenance of AF (Chappey et al., 1993).

Colchicine can exert anti-inflammatory effects by binding to free tubulin dimers, which is the basic mechanism of colchicine (Imazio and Nidorf, 2021). Low concentrations of colchicine can prevent cytoplasmic microtubule polymerization, and LoDoCo have therapeutic effects and remain effective within a few days of administration (Leung et al., 2015). High concentrations of colchicine can promote microtubule depolymerization, thereby preventing immune cell activation and reducing the inflammatory response (Chaldakov and Vankov, 1986). Originally described in arterial smooth muscle cells, microtubules have been found to be closely associated with organelles involved in protein synthesis, particularly the Golgi apparatus, especially in vascular smooth muscle cells of the synthetic phenotype. (Bhattacharyya et al., 2008). Since then, there has been increasing evidence that microtubules are important components of many cytoskeletons, participate in intracellular transport activities, affect cytokine and chemokine release, and control ion channels and cell division (Andreu and Timasheff, 1982; Van Wagoner, 2011). Colchicine forms a soluble complex with tubulin with high affinity, which affects the formation of microtubules (Martinon et al., 2006; Van Wagoner, 2011).

Thus, colchicine can directly reduce inflammation by blocking inflammatory signalling networks (Bonaventura and Montecucco, 2019). Colchicine mainly changes the adhesion of endothelial cells and leukocytes, thereby inhibiting the activation, recruitment and migration of neutrophils (Chia et al., 2008; Terkeltaub, 2009). Superoxide is an important factor in neutrophil activation, and colchicine inhibits superoxide production (Cronstein et al., 1995). Colchicine also reduces the expression of E-selectin in endothelial cells and L-selectin in neutrophils, which affects the recruitment of neutrophils (Asako et al., 1992). Furthermore, colchicine reduces platelet activating factor (PAF) and leukotriene-β4 (LT-β4), which weakens the induction of neutrophil adhesion and inhibits neutrophil migration (Dalbeth et al., 2014). In addition, colchicine attenuates vesicle trafficking, reduces the expression of TNF-α receptors on macrophages and blocks mast cell degranulation (Marques-da-Silva et al., 2011). Colchicine was shown to reduce the levels of the proinflammatory cytokines IL-1β, IFN-γ, IL-18, and IL-6 *in vivo* and *in vitro* (Martínez et al., 2018).

The NOD-like receptor pyrin containing domain 3 (NLRP3) inflammasome is associated with the antiinflammatory effects of colchicine. Colchicine affects the activity of NLRP3, which decreases the release of IL-1 and IL-18 (Yao et al., 2018). The impact of NLRP3 was amplified in the atrial cardiomyocytes of an AF model (Triantafilou et al., 2016). NLRP3 is a cytoplasmic complex present in neutrophils, monocytes, eosinophils and cardiomyocytes (Martinon et al., 2009; Buckley and Abbate, 2018). The NLRP3 inflammasome consists of the Toll-like receptor NLRP3, adaptor protein apoptosis-related spot-like protein (ASC) and the cysteine protease caspase-1 (Campbell et al., 2016). Inflammasome activation triggers an increase in the expression of inflammatory components, which in turn widely stimulates the activation of caspase-1 and produces the activated inflammatory factors interleukin-1β (IL-1β) and IL-18, which are important mediators of the inflammatory cascade (Schenone and Menon, 2018; D'Amario et al., 2021). By blocking activation of the NLRP3 inflammasome, colchicine blocks this activation process and inhibits the release of IL-1 and IL-18 (Martínez et al., 2015; Fiolet et al., 2020). Research has shown that short-term colchicine treatment in ACS patients significantly reduces the levels of inflammatory markers such as IL-1, IL-18, and IL-6 (Martinon et al., 2002; Bhattacharyya et al., 2008; Otani et al., 2016). According to recent research, colchicine can inhibit NLRP3 activation in the following ways. Colchicine can prevent the MEFV gene from being expressed, which prevents the production of NLRP3 (Li et al., 1999; Misawa et al., 2013; Nidorf et al., 2014). In addition, colchicine can inhibit pore formation, resulting in a decrease in intracellular K^+^, thereby reducing ROS and IL-1β levels (Martínez et al., 2018). Nidoret et al. found that colchicine decreased the risk of cholesterol crystallization and disrupted neutrophil function, thereby preventing related inflammatory responses (Misawa et al., 2013).

### 5.2 Colchicine affects atrial structural remodelling

Atrial fibrosis can slow the conduction of local areas, resulting in increased heterogeneity of conduction, which in turn contributes to arrhythmia (Goette et al., 2002). The mechanism and clinical importance of atrial structural remodelling have been supported by numerous investigations. Left atrial dilatation and left atrial fibrosis can lead to atrial structural remodelling. In AF patients, one of the main causes of cardiac remodelling is the development of myocardial fibrosis (Nikolaidis et al., 2006; Galea et al., 2014).

Previous studies have suggested that fibrosis in atherosclerosis and hypertension may be based on the coupling between the microtubule cytoskeleton and protein secretion, and people have begun to look for ways to control fibrosis using microtubules as an entry point (Bhattacharyya et al., 2008). Colchicine has potential as a drug that can control microtubule polymerization. The exact mechanism of the antifibrotic effect of colchicine is still unclear, but its effect has been confirmed in multiple models (Clare and Troughton, 2007; Wu et al., 2020). Colchicine prevents AF in SP rats by inhibiting atrial fibrosis (Rennard et al., 1988). In an *in vivo* model, colchicine exerted an indirect antifibrotic effect by inhibiting the release of profibrotic factors (Singhal et al., 2014). In a rabbit model of heart failure, colchicine reduced left atrial fibrosis (Yue et al., 2019). Studies have shown that colchicine has acute cardiac protective effects and a positive effect on cardiac haemodynamics. After the application of colchicine, left ventricular remodelling was reduced, and the commonly used parameters for evaluating left ventricular diameter (LVEF) were improved (Bresson et al., 2021). Transcriptomics analysis showed that the mechanism of colchicine involved a pathway closely related to myocardial fibrosis (Yue et al., 2022). Current research shows that colchicine can inhibit the TGFβ1/ALK5 pathway and activin A/ALK4 fibrosis pathways, thereby exerting an antifibrotic effect (Christ et al., 2004).

### 5.3 Colchicine affects electrical remodelling

AF is related to the electrical remodelling of cardiac ion channels, which include Ca^2+^ and K^+^ channels (Nattel et al., 2007; Nattel et al., 2020). AF is caused by ectopic activity, which is the spontaneous depolarization of atrial tissue away from the sinus node (Zimetbaum, 2017). Studies have confirmed that its main mechanism involves changing the function of the ryanodine receptor 2 (RyR2) Ca^2+^ release channel and ion channel so that the diastolic sarcoplasmic reticulum (SR) releases more Ca^2+^, thereby shortening the duration of the action potential (Workman et al., 2006). Ion channels also play a role in POAF. According to previous studies, people with POAF had more L-type Ca^2+^ channels in their atrial cardiomyocytes, which is indicative of the role excess calcium plays in AF (Van Wagoner et al., 1999; Swartz et al., 2009). In POAF patients and non-POAF patients, K^+^ channel transcripts were not significantly changed (Kerfant et al., 2001).

Colchicine directly regulates calcium (Ca^2+^) homeostasis in cardiomyocytes (137). Colchicine can weaken sympathetic nerve activity and responses; furthermore, it can phosphorylate Ca^2+^ ion channels, which can reduce calcium overload, reduce ectopic activity, and thus reduce the possibility of inducing AF (Andreu and Timasheff, 1982).

## 6 Future prospects

**FIGURE 1:**
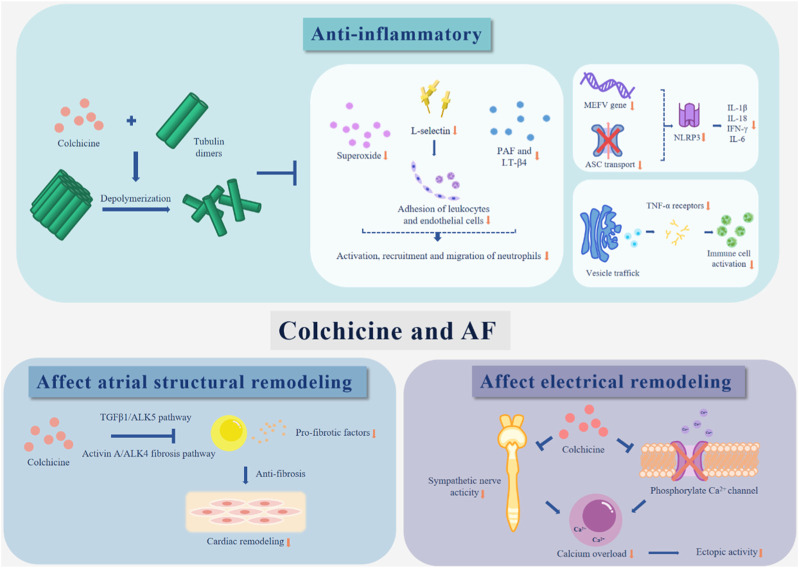
The possible mechanisms of colchicine in the treatment of AF.

Colchicine has good application prospects in clinical practice. Its preventive and therapeutic effects on AF have been found in many studies. This article summarized the possible mechanisms of colchicine in the treatment of AF (Table 2; Figure 1), but its specific mechanism has not been clearly determined and needs further study. Furthermore, considering that there are many cases of drug withdrawal due to adverse reactions in clinical trials, future research is required to determine the timing and dose of colchicine that will have the greatest effectiveness and tolerance to further improve the medication regimen. In addition, colchicine has been examined for its ability to prevent AF in people who have undergone cardiac surgery. Future confirmatory studies on the efficacy, safety, and precise timing of colchicine treatment to avoid POAF are urgently needed. In summary, more exploration is needed before colchicine is fully applied in clinical practice.

**TABLE 2:** Research on the mechanism of colchicine in treating AF.

First author	Year	Study model	Key conclusion
Kerfant et al. (2001)	2001	Rat ventricular myocytes	Colchicine can regulate Ca^2+^ signal transduction in cardiomyocytes by disrupting microtubules
Cronstein et al. (1995)	2008	*In vivo* model of acute gout inflammation in humans and mice	Colchicine at subtoxic doses can inhibit superoxide production *in vivo*
Martínez et al. (2018)	2011	Fresh or cultured mouse peritoneal macrophages	The production of ROS, IL-1β, IFN-γ and NO decreased after colchicine treatment
Yue et al. (2019)	2014	Rabbit heart failure model	Colchicine can activate PI3K/Akt/eNOS signalling pathway, reverse atrial remodelling and inhibit AF.
Nidorf et al. (2014)	2016	Small intestine injury mouse model	Colchicine inhibited activation of the NLRP3 inflammasome and reduced the protein expression of caspase-1 and IL-1β, but did not affect the mRNA expression of NLRP3 or IL-1β
Yue et al. (2022)	2019	Rat AF model	The effect of colchicine on AF involves the AKAP4, Pcdha9, GP2, CD177, Krt15, Aqp3, Chia and Bpifb1 genes, and related pathways
Christ et al. (2004)	2022	Rat AF model	Colchicine can treat AF by inhibiting the fibrosis-associated TGFβ1/ALK5 and activin A/ALK4 pathways
